# Human Papillomavirus Vaccination Coverage Among Adolescent Girls, 2007–2012, and Postlicensure Vaccine Safety Monitoring, 2006–2013 — United States

**Published:** 2013-07-26

**Authors:** Shannon Stokley, C. Robinette Curtis, Jenny Jeyarajah, Theresa Harrington, Julianne Gee, Lauri Markowitz

**Affiliations:** Immunization Services Div, National Center for Immunization and Respiratory Diseases; Immunization Safety Office, National Center for Emerging, Zoonotic, and Infectious Diseases; Div of Sexually Transmitted Diseases, National Center for HIV/AIDS, Viral Hepatitis, STD, and TB Prevention, CDC

Since mid-2006, the Advisory Committee on Immunization Practices (ACIP) has recommended routine vaccination of adolescent girls at ages 11 or 12 years with 3 doses of human papillomavirus (HPV) vaccine (1). Two HPV vaccines are currently available in the United States. Both the quadrivalent (HPV4) and bivalent (HPV2) vaccines protect against HPV types 16 and 18, which cause 70% of cervical cancers and the majority of other HPV-associated cancers; HPV4 also protects against HPV types 6 and 11, which cause 90% of genital warts.* This report summarizes national HPV vaccination coverage levels among adolescent girls aged 13–17 years† from the 2007–2012 National Immunization Survey-Teen (NIS-Teen) and national postlicensure vaccine safety monitoring. Although vaccination coverage with ≥1 dose of any HPV vaccine increased from 25.1% in 2007 to 53.0% in 2011, coverage in 2012 (53.8%) was similar to 2011. If HPV vaccine had been administered during health-care visits when another vaccine was administered, vaccination coverage for ≥1 dose could have reached 92.6%. Safety monitoring data continue to indicate that HPV4 is safe. Despite availability of safe and effective vaccines and ample opportunities for vaccine delivery in the health-care setting, HPV vaccination coverage among adolescent girls failed to increase from 2011 to 2012.

## Vaccination Coverage

Since 2006, NIS-Teen has collected vaccination information for adolescents aged 13–17 years in the 50 states, the District of Columbia, and selected areas,§ using a random-digit–dialed sample of landline and (starting in 2011) cellular telephone numbers.¶ After a teen’s parent/guardian grants permission to contact their teen’s vaccination provider(s), a questionnaire is mailed to each provider to obtain a vaccination history from medical records. In 2012, the Council of American Survey Research Organizations (CASRO) landline response rate was 55.1%. A total of 14,133 adolescents with vaccination provider–reported vaccination records were included, representing 62% of all adolescents from the landline sample with completed household interviews. The cellular telephone sample CASRO response rate was 23.6%. A total of 5,066 adolescents with vaccination provider–reported vaccination records were included, representing 56.4% of all adolescents from the cellular telephone sample with completed household interviews.** Analysis for this report was limited to girls with provider-reported vaccination histories.†† HPV vaccination coverage represents receipt of any HPV vaccine and does not distinguish between HPV2 or HPV4. NIS-Teen methodology, including weighting procedures, has been described previously.§§ Differences in vaccination coverage were evaluated using t-tests and were considered statistically significant if p≤0.05.

Vaccination coverage was assessed for each dose of the HPV vaccination series: ≥1 dose represents initiation of the series, ≥2 doses represents progress with girls returning for additional doses, and ≥3 doses represents completion of the series. Coverage for ≥1, ≥2, and ≥3 HPV doses significantly increased annually during 2007–2011, but 2011 and 2012 coverage levels were similar (Table 1).

A missed opportunity was defined as a health-care encounter occurring on or after a girl’s 11th birthday and on or after March 23, 2007 (the publication date of ACIP’s HPV4 recommendation), during which a girl received at least one vaccine but did not receive HPV vaccine. The percentage of unvaccinated girls with at least one missed opportunity for HPV vaccination increased from 20.8% in 2007 to 84.0% in 2012 (Table 1). In 2012, if all missed opportunities for HPV vaccination had been eliminated, coverage with ≥1 dose of HPV vaccine could have reached 92.6% (Table 1).

The 2012 NIS-Teen asked parents who did not intend to vaccinate their daughters in the next 12 months (23% of respondents) the main reason why their daughters would remain unvaccinated. The top five responses were as follows: vaccine not needed (19.1%), vaccine not recommended (14.2%), vaccine safety concerns (13.1%), lack of knowledge about the vaccine or the disease (12.6%), and daughter is not sexually active (10.1%).

## Vaccine Safety

In the United States, postlicensure vaccine safety monitoring and evaluation are conducted independently by federal agencies and vaccine manufacturers. From June 2006 through March 2013, approximately 56 million doses of HPV4 were distributed in the United States, and from October 2009 through May 2013, a total of 611,000 doses of HPV2 were distributed. Because HPV4 accounts for 99% of the doses distributed in the United States, analysis of vaccine safety data was limited to HPV4. During June 2006–March 2013, the Vaccine Adverse Event Reporting System (VAERS)¶¶ received a total of 21,194 adverse event reports occurring in females after receipt of HPV4; 92.1% were classified as nonserious. Reporting peaked in 2008 and decreased each year thereafter; the proportion of reports to VAERS that were classified as serious reports*** peaked in 2009 at 12.8% and decreased thereafter to 7.4% in 2013 (Figure). Among nonserious adverse events, the most commonly reported generalized symptoms were syncope (fainting), dizziness, nausea, headache, fever, and urticaria (hives); the most commonly reported local symptoms were injection-site pain, redness, and swelling. Among the 7.9% of HPV4-related VAERS reports classified as serious, headache, nausea, vomiting, fatigue, dizziness, syncope, and generalized weakness were the most frequently reported symptoms. Overall reporting of adverse events to VAERS is consistent with prelicensure clinical trial data and, during the last 7 years, reporting patterns have remained consistent with the 2009 published summary of the first 2.5 years of postlicensure reporting to VAERS (2).

Three population-based published studies of HPV4 vaccine safety, including one from CDC’s Vaccine Safety Datalink,††† have been conducted in the United States (Table 2). Although one postlicensure observational study found an increased risk for syncope, no serious safety concerns have been identified in these large postlicensure observational studies.

### Editorial Note

Although HPV vaccination coverage has lagged behind that of other vaccines recommended for adolescents (3), coverage among adolescent girls increased each year during 2007–2011; 2012 is the first year with no observed increase. In 2012, only 53.8% of girls had received ≥1 dose of HPV vaccine, and only 33.4% had received all 3 doses of the series. Despite the availability of safe and effective HPV vaccines, approximately one quarter of surveyed parents did not intend to vaccinate their daughters in the next 12 months. Missed vaccination opportunities remain high. Every health-care visit, whether for back-to-school evaluations or acute problems, should be used to assess teenagers’ immunization status and provide recommended vaccines if indicated.

Approximately 79 million persons in the United States are infected with HPV, and approximately 14 million will become newly infected each year (4). Some HPV types can cause cervical, vaginal, and vulvar cancer among women; penile cancer among men; and anal and some oropharyngeal cancers among both men and women (4). Other HPV types can cause genital warts among both sexes (4). Each year in the United States, an estimated 26,200 new cancers attributable to HPV occur: 17,400 among females (of which 10,300 are cervical cancer) and 8,800 among males (of which 6,700 are oropharyngeal cancers).§§§

What is already known on this topic?

Since mid-2006, a licensed human papillomavirus (HPV) vaccine has been available and recommended by the Advisory Committee on Immunization Practices for routine vaccination of girls at ages 11 or 12 years. Based on results of the 2011 National Immunization Survey-Teen, only 53.0% of girls aged 13–17 years received ^3^1 dose of HPV vaccine, and only 34.8% received all 3 doses of the HPV vaccine series.

What is added by this report?

Vaccination coverage of adolescent girls remained unchanged in 2012; only 53.8% of girls received ^3^1 dose of HPV vaccine, and only 33.4% received all 3 doses of the series. Among unvaccinated girls, 84% had a health-care encounter in which they received a vaccine but not HPV vaccine. National safety monitoring data continue to indicate that the quadrivalent HPV vaccine is safe.

What are the implications for public health practice?

Despite the availability of safe and effective vaccines, many girls remain unprotected against HPV infections. If HPV vaccine was administered at health-care encounters when other recommended vaccines were administered, vaccination coverage could be as high as 92.6%. Improving practice patterns so that health-care providers and their staff members use every opportunity to offer HPV vaccines and are well-equipped to address questions from parents is necessary to reduce HPV-attributable cancers further.

Because cancers attributable to HPV occur years after infection, decades might be required before the impact of vaccination on reducing cancers is well-documented. However, shorter-term, vaccine-preventable outcomes are being monitored (including HPV prevalence, genital warts, and cervical precancers). Recent data from the National Health and Nutrition Examination Survey show a greater than 50% decrease in HPV infections caused by types targeted by HPV4 vaccine among females aged 14–19 years within the first 4 years of the HPV vaccination program (5). Administrative claims data from privately insured patients show declining genital warts incidence among patients aged 15–19 years, from 2.9 per 1,000 person-years in 2006 to 1.8 in 2010 (6). Substantial reductions in genital warts have occurred in other countries where vaccination programs achieved high coverage in target and catch-up age groups (7,8). In Australia, where the national vaccination program targeted females, rates of genital warts also decreased among males (7).

In addition to prelicensure HPV4 clinical trials that demonstrated safety and efficacy among thousands of patients, nearly 7 years of postlicensure vaccine safety monitoring provide further evidence of the safety of HPV4. Syncope can occur among adolescents who receive vaccines, including HPV4. To decrease the risk for falls and other injuries that might follow syncope, ACIP recommends that clinicians consider observing patients for 15 minutes after vaccination.

This report highlights three areas that need to be addressed to improve HPV vaccination coverage. The first area is education of parents. Three of the five main reasons parents reported for not intending to vaccinate their daughters (i.e., vaccine not needed, lack of knowledge, and daughter not sexually active) indicate gaps in understanding, including why vaccination is recommended by age 13 years. Parents also reported vaccine safety concerns as a main reason for not vaccinating. Updated educational materials that address these issues are available from CDC at http://www.cdc.gov/vaccines/who/teens/index.html.

Second, health-care providers must increase the consistency and strength of HPV vaccination recommendations. Studies have documented that, especially when counseling younger adolescents or their parents, providers give weaker recommendations for HPV vaccination compared with other vaccinations recommended for adolescents (9). Because provider counseling and recommendations greatly influence parental acceptance of vaccines, CDC has recently developed a tip sheet (available at http://www.cdc.gov/vaccines/who/teens/for-hcp-tipsheet-hpv.html) to help providers respond to parents’ questions and communicate strong, clear HPV vaccination recommendations.

Finally, missed vaccination opportunities need to be reduced. Although providers cite infrequent preventive health-care visits among the adolescent population as a vaccination barrier (10), these data demonstrate that health-care access is not the main impediment. The increase in missed opportunities observed during 2007–2012 is attributable to higher and steadily increasing coverage for other vaccines recommended for adolescents (3). The 2012 NIS-Teen shows that 84% of unvaccinated girls had a health-care encounter where another vaccine was administered. Had the 3-dose HPV series been initiated at these visits, coverage for ≥1 dose could be as high as 92.6%.

High HPV vaccination coverage with existing infrastructure and health-care utilization is possible in the United States. Taking advantage of every health-care encounter, including acute-care visits, to assess every adolescent’s vaccination status can help minimize missed opportunities. Potential strategies include using vaccination prompts available through electronic health records or checking local and state immunization information systems to assess vaccination needs at every encounter. Series completion also can be promoted through scheduling appointments for second and third doses before patients leave providers’ offices after receipt of their first HPV vaccine doses and with automated reminder-recall systems.

The findings in this report are subject to at least four limitations. First, the cellular telephone household response rate was only 23.6%, and the landline household response rate was only 56.1%. Nonresponse and noncoverage bias (from exclusion of households without telephones) might remain after weighting adjustments. Second, underestimates of vaccination coverage might have resulted from the exclusive use of provider-verified vaccination histories because the completeness of the records is unknown. Third, frequency of missed opportunities might be underestimated because health-care encounters in which a vaccination was not administered could not be included. Finally, VAERS is a passive reporting system that accepts reports from anyone, including health-care providers, patients, or family members. VAERS cannot determine cause-and-effect; a report of an adverse event to VAERS does not mean that a vaccine caused the event. Underreporting might occur and serious medical events are more likely to be reported than minor ones.

Additional information on VAERS is available at http://vaers.hhs.gov/data/index. The Vaccine Safety Datalink (VSD) is a population-based monitoring system that evaluates adverse events in those vaccinated with HPV vaccine compared with a control group and can estimate risk. Safety concerns raised through VAERS are evaluated more thoroughly using VSD. Data from VSD and from other published population-based studies provide more specific evidence about vaccine safety.

By increasing 3-dose HPV vaccination coverage to 80%, an estimated additional 53,000 cases of cervical cancer could be prevented over the lifetimes of those aged ≤12 years.¶¶¶ For every year that increases in coverage are delayed, another 4,400 women will go on to develop cervical cancer. Improving practice patterns and clinical skills so that health-care providers are well-equipped to address questions from parents and are committed to using every opportunity to strongly recommend HPV vaccination is necessary to achieve potential reductions in HPV-attributable cancers.

## Figures and Tables

**FIGURE:**
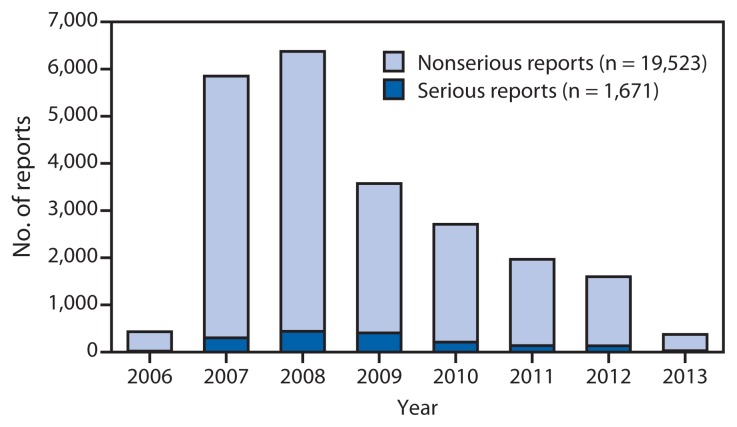
Number of serious and nonserious reports of adverse events after administration of quadrivalent human papillomavirus (HPV4) vaccine in females, by year — Vaccine Adverse Event Reporting System, United States, June 2006–March 2013* * Total number of reports (serious and nonserious) = 21,194. In the Vaccine Adverse Event Reporting System, reports are classified as serious if the submitter reports one or more of the following: hospitalization, prolongation of an existing hospitalization, permanent disability, life-threatening illness, or death.

**TABLE 1:** Estimated human papillomavirus (HPV) vaccine coverage among adolescent girls aged 13–17 years, by number of doses — National Immunization Survey–Teen, United States, 2007–2012

Characteristic	Survey year*											

	2007		2008		2009		2010		2011		2012	
					
	%	(95% CI)	%	(95% CI)	%	(95% CI)	%	(95% CI)	%	(95% CI)	%	(95% CI)
≥1 dose HPV vaccine†	25.1	(22.3–28.1)	37.2	(35.2–39.3)§	44.3	(42.4–46.1)§	48.7	(46.9–50.5)§	53.0	(51.4–54.7)§	53.8	(52.0–55.7)
≥2 doses HPV vaccine	16.9	(14.6–19.6)	28.3	(26.4–30.3)§	35.8	(34.1–37.6)§	40.7	(38.9–42.5)§	43.9	(42.3–45.6)§	43.4	(41.5–45.2)
≥3 doses HPV vaccine	5.9	(4.4–7.8)	17.9	(16.3–19.6)§	26.7	(25.2–28.3)§	32.0	(30.3–33.6)§	34.8	(33.2–36.4)§	33.4	(31.7–35.2)
Unvaccinated girls with ≥1 missed opportunity for HPV vaccine¶	20.8	(17.6–24.3)	30.8	(28.5–33.2)§	52.5	(50.1–55.0)§	67.9	(65.5–70.2)§	77.7	(75.7–79.6)§	84.0	(82.1–85.8)§
Potential coverage with ≥1 dose of HPV vaccine if no missed opportunity	40.6	(37.3–44.0)	56.5	(54.4–58.6)§	73.5	(71.9–75.1)§	83.5	(82.2–84.8)§	89.5	(88.5–90.5)§	92.6	(91.7–93.5)§

**Abbreviation:** CI = confidence interval.

* The number of adolescent girls with provider-reported vaccination histories for each survey year are as follows: 2007, n = 1,440; 2008, n = 8,607; 2009, n = 9,621; 2010, n = 9,220; 2011, n = 11,236; and 2012, n = 9,058.

† HPV, either quadrivalent or bivalent.

§ Statistically significant difference (p≤0.05) compared with the previous year’s estimate.

¶ Missed opportunity defined as a health-care encounter occurring on or after a girl’s 11th birthday and on or after March 23, 2007 (the publication date of the Advisory Committee on Immunization Practices’ HPV4 recommendation), during which a girl received at least one vaccine but did not receive HPV vaccine.

**TABLE 2:** Published population-based, postlicensure observational safety studies of HPV4 vaccine in U.S. females aged 9–26 years

Organization	System or review	No. of doses evaluated	Description	Methods	Findings
CDC	Vaccine Safety Datalink*	600,559	Large database used for active surveillance and research; safety assessment of seven prespecified health outcomes among female HPV4 vaccine recipients at seven managed-care organizations†	Cohort design with weekly sequential analyses of electronic medical data§	No statistically significant increase in risk for the outcomes monitored
Merck	Postmarketing commitment to FDA¶	346,972	General study assessment of HPV4 vaccine after routine administration at two large managed-care organizations	Self-controlled risk interval design, supplemented with medical record review	HPV4 vaccine associated with syncope on the day of vaccination and skin infections** in the 2 weeks after vaccination; no other vaccine safety signals detected
Merck	Postmarketing commitment FDA††	346,972	Assessment of 16 prespecified autoimmune conditions after routine use of HPV4 vaccine at two large managed-care organizations	Retrospective cohort using electronic medical data, supplemented with medical record review§§	No confirmed safety signals for the outcomes monitored

**Abbreviations:** HPV4 = quadrivalent human papillomavirus; FDA = Food and Drug Administration.

* Gee J, Naleway A, Shui I, et al. Monitoring the safety of quadrivalent human papillomavirus vaccine: findings from the Vaccine Safety Datalink. Vaccine 2011;29:8270–84.

† Prespecified outcomes included Guillain-Barré syndrome, stroke, appendicitis, seizures, allergic reactions, anaphylaxis, syncope, and venous thromboembolism

§ Comparison groups included historic background rates for Guillain-Barré syndrome, stroke, appendicitis, venous thromboembolism, and anaphylaxis; concurrent preventive health visits for seizures; or adolescent vaccination visits for syncope and allergic reactions.

¶ Klein NP, Hansen J, Chao C, et al. Safety of quadrivalent human papillomavirus vaccine administered routinely to females. Arch Pediatr Adolesc Med 2012; 166:1140–8.

** Medical record review suggested some cases might have been local injection site reactions.

†† Chao C, Klein NP, Velicer CM, et al. Surveillance of autoimmune conditions following routine use of quadrivalent human papillomavirus vaccine. J Intern Med 2012;271:193–203.

§§ Comparison group included background incidence rates.

## Footnotes

* Quadrivalent HPV vaccine was licensed in 2006 (information available at http://www.cdc.gov/mmwr/preview/mmwrhtml/rr5602a1.htm) and the bivalent HPV vaccine was licensed in 2009 (information available at http://www.cdc.gov/mmwr/preview/mmwrhtml/mm5920a4.htm?s_cid=mm5920a4_e).

† For each survey year, eligible participants were born during the following periods: 2007, October 1989–February 1995; 2008, January 1990–February 1996; 2009, January 1991–February 1997; 2010, January 1992–February 1998; 2011, January 1993–February 1999; and 2012, January 1994–February 2000.

§ Six areas that received federal Section 317 immunization grants were sampled separately: District of Columbia; Chicago, Illinois; New York, New York; Philadelphia County, Pennsylvania; Bexar County, Texas; and Houston, Texas.

¶ All identified cellular telephone households from the cellular telephone sampling frame were eligible for interview; sampling weights have been adjusted from dual-frame sampling, nonresponse, noncoverage, and overlapping samples of mixed telephone users (i.e., those having both a landline and a cellular telephone). A description of NIS-Teen dual-frame survey methodology and its effect on reported vaccination estimates is available at http://www.cdc.gov/vaccines/stats-surv/nis/dual-frame-sampling-08282012.htm.

** The CASRO response rate is the product of three other rates: 1) the resolution rate, which is the proportion of telephone numbers that can be identified as either for a business or residence; 2) the screening rate, which is the proportion of qualified households that complete the screening process; and 3) the cooperation rate, which is the proportion of contracted eligible households for which a completed interview is obtained. CASRO response rates for survey years 2007–2011 are available at ftp://ftp.cdc.gov/pub/health_statistics/nchs/dataset_documentation/nis/nisteenpuf11_dug.pdf.

†† The number of adolescent girls with provider-reported vaccination histories for each survey year are as follows: 2007, n = 1,440; 2008, n = 8,607; 2009, n = 9,621; 2010, n = 9,220; 2011, n = 11,236; 2012, n = 9,058.

§§ Information available at ftp://ftp.cdc.gov/pub/health_statistics/nchs/dataset_documentation/nis/nisteenpuf10_codebook.pdf.

¶¶ Additional information available at http://www.cdc.gov/vaccinesafety/activities/vaers.html.

*** In VAERS, reports are classified as serious if the submitter reports one or more of the following: hospitalization, prolongation of an existing hospitalization, permanent disability, life-threatening illness, or death.

††† Additional information available at http://www.cdc.gov/vaccinesafety/activities/vsd.html.

§§§ Additional information available at http://www.cdc.gov/cancer/hpv/statistics/cases.htm.

¶¶¶ Estimates obtained from an adaptation of the model presented in the following report: Chesson HW, Ekwueme DU, Saraiya M, Dunne EF, Markowitz LE. The cost-effectiveness of male HPV vaccination in the United States. Vaccine 2011;29:8443–50.
